# Development, testing and validation of a SARS-CoV-2 multiplex panel for detection of the five major variants of concern on a portable PCR platform

**DOI:** 10.3389/fpubh.2022.1042647

**Published:** 2022-12-15

**Authors:** Bryce J. Stanhope, Brittany Peterson, Brittany Knight, Ray Nobles Decadiz, Roger Pan, Phillip Davis, Anne Fraser, Manunya Nuth, Jesse vanWestrienen, Erik Wendlandt, Bruce Goodwin, Christopher Myers, Jennifer Stone, Shanmuga Sozhamannan

**Affiliations:** ^1^Biomeme, Inc., Philadelphia, PA, United States; ^2^MRIGlobal, Kansas City, MO, United States; ^3^Naval Health Research Center (NHRC), San Diego, CA, United States; ^4^Integrated DNA Technologies, Coralville, IA, United States; ^5^Defense Biological Product Assurance Office (DBPAO), Joint Program Executive Office for Chemical, Biological, Radiological and Nuclear Defense (JPEO-CBRND), Enabling Biotechnologies, Frederick, MD, United States; ^6^Logistics Management Institute, Tysons, VA, United States

**Keywords:** SARS-CoV-2, variants, PCR, Biomeme, rapid detection, screening assay, RUO

## Abstract

Many SARS-CoV-2 variants have emerged during the course of the COVID-19 pandemic. These variants have acquired mutations conferring phenotypes such as increased transmissibility or virulence, or causing diagnostic, therapeutic, or immune escape. Detection of Alpha and the majority of Omicron sublineages by PCR relied on the so-called S gene target failure due to the deletion of six nucleotides coding for amino acids 69–70 in the spike (S) protein. Detection of hallmark mutations in other variants present in samples relied on whole genome sequencing. However, whole genome sequencing as a diagnostic tool is still in its infancy due to geographic inequities in sequencing capabilities, higher cost compared to other molecular assays, longer turnaround time from sample to result, and technical challenges associated with producing complete genome sequences from samples that have low viral load and/or high background. Hence, there is a need for rapid genotyping assays. In order to rapidly generate information on the presence of a variant in a given sample, we have created a panel of four triplex RT-qPCR assays targeting 12 mutations to detect and differentiate all five variants of concern: Alpha, Beta, Gamma, Delta, and Omicron. We also developed an expanded pentaplex assay that can reliably distinguish among the major sublineages (BA.1–BA.5) of Omicron. *In silico*, analytical and clinical testing of the variant panel indicate that the assays exhibit high sensitivity and specificity. This panel can help fulfill the need for rapid identification of variants in samples, leading to quick decision making with respect to public health measures, as well as treatment options for individuals. Compared to sequencing, these genotyping PCR assays allow much faster turn-around time from sample to results—just a couple hours instead of days or weeks.

## Background

The emergence of COVID-19 in late 2019 in Wuhan, China and subsequent spread of the virus across the globe led to the unprecedented COVID-19 pandemic and declaration as a Public Health Emergency of Global Concern on Jan 30, 2020 by the World Health Organization (WHO) (1). Identification of the etiological agent of COVID-19 as SARS-CoV-2 and release of the first whole genome sequence of SARS-CoV-2 shortly thereafter on Jan 11, 2020 was instrumental in the rapid development of diagnostics, therapeutics and vaccines (2, 3). Public health measures (such as “test, trace and isolate”) and individual health measures (such as treating symptomatic individuals as needed) rely on rapid and accurate diagnosis early in the course of infection. The first molecular diagnostic test was issued an emergency use authorization (EUA) on Feb 04, 2020. As of May 05, 2022 (~2 years later), there were 307 EUA SARS-CoV-2 molecular tests and 48 antigen tests (4). The COVID-19 pandemic response has been complicated by the fact that as many as 40% of infected individuals may be asymptomatic depending on the population cohort, while secondary transmission might still be occurring (5). Hence, PCR tests remain the gold standard for SARS-CoV-2 detection because of their exquisite sensitivity and better performance identifying asymptomatic cases when compared to rapid antigen tests (6). It has been argued that PCR tests may show positivity for a longer duration—even after the individual is no longer infectious—and hence rapid antigen tests may be more reflective of infectivity (7, 8). However, PCR tests are preferred for their sensitivity, especially in testing for asymptomatic infections and early in the infection cycle (9, 10).

Despite the availability of hundreds of different tests, testing demands and needs have not been fully met and there is always a shortage of test quantities and sites around the globe. This adversely affects rapid decision making to test, trace, and isolate infected persons to prevent secondary transmissions (11). For this reason, there is a need for rapid and accurate tests that have higher sensitivity and can be performed in less complex settings. Indeed, at-home diagnostic kits meet the turn-around time requirements, but few options are currently available for sensitive PCR-based tests compared to the less sensitive rapid antigen tests (12). In addition to testing constraints, the emergence of variants has further complicated testing capabilities.

Rapid proliferation and sustained transmission of SARS-CoV-2 in the human population resulted in waves of variants that swept through the globe (13, 14). To identify the variants, genomic surveillance has come to the forefront of the COVID-19 pandemic response, as evidenced by the tremendous explosion of whole genome sequencing data from all around the globe (15). Whole genome sequencing has become ubiquitous, and as of May 24, 2022, more than 11 million SARS-CoV-2 sequences have been generated and deposited to the GISAID database by the global sequencing community (16–18). Whole genome sequence data have been extensively used in determining the changing genetic profiles of viral variants and tracking viral mobility across communities in what has come to be known as genomic epidemiology (19–21). Rapid identification of the prevalent variants without having to wait weeks for sequence data is paramount for public health measures and decision-making, and in some instances even treatment options. For example, some antibody therapies fail against Omicron infections due to specific mutations in critical positions in the spike protein (22), and antibody evasion capabilities vary not only amongst the major lineages (23), but also amongst the Omicron sublineages (24). This prompted the U.S. Food and Drug Administration to release a statement in January of 2022 that limited the use of certain antibody treatments to only patients known to be infected/exposed to susceptible lineages (25). Hence, there is a need for RT-qPCR assays that can rapidly provide genotype information just a couple hours after sample collection, compared to the many days that are usually required before sequencing results are available (26–36).

Here we describe a panel of multiplex RT-qPCR assays to detect and differentiate all five WHO designated variants of concern: Alpha, Beta, Gamma, Delta, and Omicron. These RT-qPCR assays can be performed on a hand-held, battery-operated device, the Biomeme Franklin™ three9 Real-Time PCR Thermocycler. We chose the Biomeme platform because it is portable, easy to use, battery-powered, and hence, field deployable with a minimal footprint. Biomeme also provides multiple assay formats—including lyophilized, room-temperature stable 3-well Go-Strips and 96-well Go-Plates—that can be used in both point-of-need, low-throughput devices like the Franklin^TM^, as well as in traditional lab-based, high-throughput instruments.

## Materials and methods

### Primers, probes and PCR reagents

The primer, probe, and template sequences that were designed in this study are presented in Supplementary Tables 1, 2. Reagents and assay conditions are described under different subsections below.

### Assay design for Triplexes 1–3

**Figure 1:**
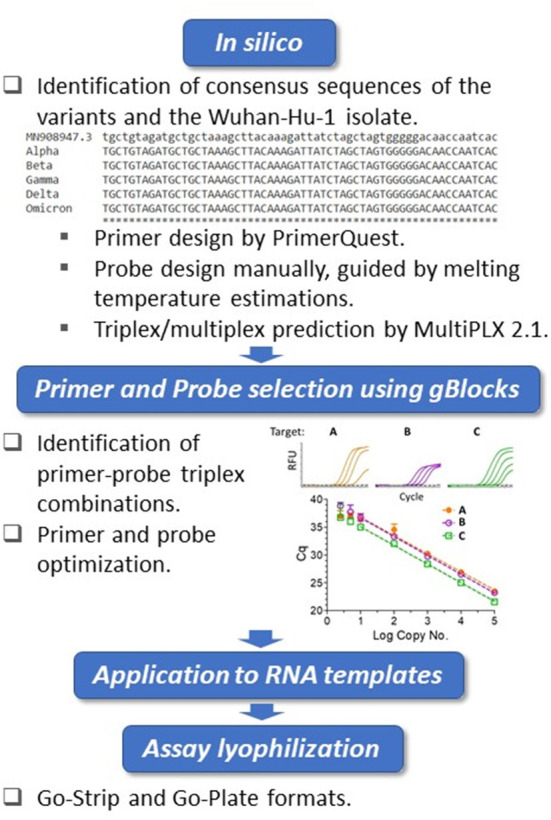
Biomeme assay design, testing and validation workflow. Triplex assay development workflow. Using the Wuhan-Hu-1 isolate (accession no. MN908947.3) as the reference, whole genomes of SARS-CoV-2 variants were compared. The *in silico* framework consisted of identification of appropriate primers and probes and their subsequent suitability in triplex combinations. The primers were designed at regions of consensus sequences, and the probes were designed manually at target mutation sites. For the locked nucleic acid (LNA) probes, melting temperature predictions served as guides for mismatch discrimination. Candidate primers and probes were then aligned using BLAST to ensure specificity, and suitable triplex combinations were subsequently predicted. Identified triplexes were then tested and optimized initially using dsDNA templates (IDT gBlocks), followed by the use of *in vitro*-transcribed RNA templates. The optimized conditions were formulated into freeze-dried Go-Strips and Go-Plates for use on the Biomeme Franklin^TM^ and standard 96-well RT-qPCR instruments.

The whole genome sequences of SARS-CoV-2 variants were downloaded from the GISAID database and aligned to the genome of the Wuhan-Hu-1 isolate (GenBank accession no. MN908947.3) using Clustal Omega (https://www.ebi.ac.uk/Tools/msa/clustalo/). Primers were designed at conserved regions of the spike (S) and nucleocapsid (N) genes to generate amplicons comprising the mutations of interest using Primer Quest Software (https://www.idtdna.com/PrimerQuest/Home/Index) from Integrated DNA Technologies, Inc. (IDT; Coralville, Iowa, USA) and analyzed for potential dimerization and hairpin products by the OligoAnalyzer tool (IDT). Probes containing LNA and conjugated minor groove binder (MGB) were manually designed. In particular, the assistance of IDT's in-house tools afforded the predication of melting temperatures and mismatch discrimination potentials. The S:Y144del primer and probe sequences were designed according to Vogels et al. (37). Primer candidates were then analyzed by Primer-BLAST (https://www.ncbi.nlm.nih.gov/tools/primer-blast/) and the probes by blastn (https://blast.ncbi.nlm.nih.gov/Blast.cgi?PAGE_TYPE=BlastSearch) to ensure recognition of the intended targets. For suitability of multiplexing, primer combinations were predicted by MultiPLX 2.1 (38). Primers and LNA probes were purchased either from IDT or Sigma-Aldrich (St. Louis, MO, USA), with the primers purified by standard desalting and the probes by high-performance liquid chromatography (HPLC). The MGB probe was purchased from Applied Biosystems (Foster City, CA, USA) as HPLC-purified products. gBlocks were purchased from IDT. An overview of the assay design and development workflow is presented in Figure 1.

### RT-qPCR on standard thermocyclers

One-step reverse transcription-quantitative polymerase chain reaction (RT-qPCR) was performed to avoid potential cross-contamination. A typical 20-μL reaction containing the DNA or RNA sample was prepared in LyoRNA 2.0 Master Mix (Biomeme, Inc., Philadelphia, PA, USA) according to the manufacturer's instructions and assayed on a Bio-Rad CFX96 instrument (Bio-Rad Laboratories, Inc., Hercules, CA, USA) in a triplex mixture. The assays were run under the following conditions: reverse transcription at 55°C for 2 min, initial denaturation at 95°C for 1 min, and 45 cycles of 95°C for 3 s and annealing/extension at 62°C for 30 s. Assay development was accomplished using gBlock templates in the RT-qPCR reactions and later confirmed with *in vitro*-transcribed RNA templates generated by the MEGAscript T7 Transcription Kit (Invitrogen, Waltham, MA, USA) according to the manufacturer's instructions using 80 ng of gBlock templates. Triplexes were tested against both wild-type and mutant templates, and only the probes that could specifically discriminate between the two templates were selected for further testing and optimization.

### RT-qPCR on the Biomeme Franklin^TM^ Thermocycler

Incorporating the same primer and probe concentrations used for measurement on the Bio-Rad CFX96 instrument, triplex assays were assembled into freeze-dried test strips (Go-Strips and Go-Plates) using Biomeme's proprietary formulations. Each well of the Go-Strip or Go-Plate was reconstituted with 20 μL total volume of nuclease-free water and template DNA/RNA. The Go-Strips were then loaded into the portable Franklin^TM^ Real-Time Thermocycler (39) in the appropriate orientation and monitored by the Biomeme software application on the included smartphone, while the Go-Plates were measured on the Bio-Rad CFX96 instrument. RT and cycling conditions used on all platforms were the same as those described for the LyoRNA master mix.

### Lyophilized assay manufacturing

Triplex assays were lyophilized into Go-Strip and Go-Plate formats. Go-Strips contained three reactions packaged individually in a small foil pouch designed for use on the Biomeme Franklin^TM^. Go-Plates are 96-well plates packaged in a foil pouch meant for standard laboratory RT-qPCR systems. Go-Strips and Go-Plates are shelf-stable at room temperatures for 18 months in their original packaging.

### Omicron assay design and optimization

Probes for the omicron multiplexes (Triplex 4 and the Omicron pentaplex) were manually designed to overlie the targeted mutation site, using Primer Express^®^ Software Version 3.0.1 to estimate melting temperatures for both the standard and MGB probes. Candidate primer pairs were designed using Primer3 Plus (40), then checked against the NCBI nt database for off-target matches and on-target mismatches using the NCBI Primer-BLAST tool (41). Candidate primer pairs that passed NCBI Primer-BLAST screening and candidate probe sequences were then tested for multiplex compatibility (oligo interactions and melting temperature) using ThermoFisher's Multiple Primer Analyzer (42), and some manual adjustments to primer sequences were made to enhance compatibility. Candidate primer and probe combinations were then screened, down-selected, and optimized using 10-fold serial dilutions of template (50–50,000 copies per reaction) to evaluate sensitivity, efficiency, and signal-to-noise ratios. Candidate assays were also tested against a high-level of wild-type genomic RNA to confirm exclusivity against non-variant template. Initial testing and optimization were performed in singleplex reactions using gBlock templates, but then progressed to multiplex testing using synthetic SARS-CoV-2 gRNA commercially available from Twist Biosciences (San Francisco, CA, USA), as well as RNA extracted from patient specimens and stock cultures (once available). Because gRNA could not be obtained for BA.1.1, BA.3, BA.4, and BA.5, gBlocks encompassing the S gene and relevant portion of the N gene were designed based on reference sequences, and serial dilutions of those were used for testing.

### Verification of assay signatures *in silico*

To evaluate inclusivity of each assay on a per-lineage basis, SARS-CoV-2 genomes designated as “complete” (*N* = 1005,125) were downloaded from NCBI Virus (43) on March 31, 2022, along with a corresponding metadata table containing the Pangolin (44) lineage assignments. Upon observing that representation of the BA.3 lineage was limited to a single record in the dataset drawn from NCBI Virus, we supplemented the dataset by downloading BA.3 assigned records (*N* = 4,370) from GISAID.org on April 15, 2022. Similarly, BA.4 sequences (*N* = 1,174) and BA.5 sequences (*N* = 663) were downloaded from GISAID.org on May 11, 2022. A complete table of author attributions for the GISAID sequences is available in Supplementary Table 3.

Inclusivity was calculated as set coverage for each assay target for each lineage. A lineage's coverage was calculated by dividing the *in silico* predicted PCR-positive sequences (based on cutoff criteria for primer and probe alignment mismatches) by the total number of sequences assigned that lineage in the database. Formally, the inclusivity *C* of each primer set is: $C=\frac{\left|B\cap A\right|}{\left|A\right|}$, where *B* is the set of records returned by a primer pair/probe and *A* is the set of records for a given lineage.

*In silico* prediction of amplicon products for the evaluated assays was conducted using a modified version of simulate_PCR (45) re-implemented in Python. This approach relies on BLAST results of the primers along with pre-specified cutoff criteria for amplicon product sizes and mismatch criteria to aggregate candidate PCR products from a target database. This pipeline was modified to better accommodate the use of short probe sequences that may contain modifications such as minor groove binders by decomposing the problem into a BLAST-based estimate of amplicon products returned by the primers, followed by a Smith-Waterman local alignment of the probe sequences against the predicted amplicon products. Because of the computational cost of alignment with expanding degeneracies, predicted amplicons that contain “N” degenerate characters are not aligned and not considered as inclusive hits. Probe alignments that meet the designated mismatch threshold are annotated to the corresponding amplicon product. This amplicon table is then evaluated using a custom Python script for inclusivity by the criteria above, where inclusivity for a set of oligos is defined as a forward primer, reverse primer, and probe sequence are all aligned within the pre-specified mismatch cutoffs. The output from this script is a summary table that gives inclusivity estimates for each unique label, in this case, the Pangolin lineage. Cutoff criteria set inclusion for all assays was a maximum of 3 mismatches in either the forward or reverse primer, 3 mismatches for standard (non-LNA) probes, and a 0 mismatch tolerance for LNA probes.

### MRIGlobal analytical testing

Assay inclusivity/exclusivity testing for all triplexes was performed using serial dilutions of RNA extracted from live SARS-CoV-2 viral stocks, whereas Omicron pentaplex testing was performed using synthetic gRNA from Twist Biosciences due to lack of available stocks for Omicron sublineages. Viral stocks were extracted using the Qiagen Viral RNA Mini Kit (Qiagen, MD, USA). A 10-fold dilution series of each extract was made and then screened for quality/quantity using the Biomeme SARS-CoV-2 Dx assay on a Bio-Rad CFX96 PCR instrument. Dilutions producing Cq values in the early 20s to late 30s (or negative results) were selected for further testing with the variant multiplexes, side-by-side with the Biomeme SARS-CoV-2 Dx assay as a control/comparator assay. This testing was performed using lyophilized versions of Triplexes 2 and 3, but “wet” versions of Triplex 1, Triplex 4, and the Omicron pentaplex were used because final lyophilized versions were not available. “Wet” versions were prepared using commercially available frozen master mix (TaqPath 1-Step RT-qPCR Master Mix, CG from ThermoFisher (St. Louis, MO, USA). When “wet” versions were used, the thermocycling protocol changed to the following: UNG incubation at 25°C for 2 min, reverse transcription at 50°C for 15 min, initial denaturation at 95°C for 2 min, and 45 cycles of 95°C for 3 s and annealing/extension at 62°C for 30 s. In addition to testing the full dilution series on the Bio-Rad CFX96, single-level testing of each extract was also performed on the handheld Biomeme Franklin^TM^ PCR device. Viral copy numbers for gRNA dilutions were later determined by droplet digital PCR (ddPCR) on a Bio-Rad QX200 (Hercules, CA, USA) and CDC 2019-nCov N1 assay primers and probe.

### MRIGlobal testing of RADx residual clinical specimens

Twenty SARS-CoV-2 residual clinical specimens were provided by the National Institutes of Health (NIH) Rapid Acceleration of Diagnostics (RADx^®^) program. These samples consisted of heat inactivated nasopharyngeal swabs in transport media that had previously tested positive for SARS-CoV-2 and had been sequenced to determine lineage before being provided to us for this study. Five samples each of four different lineages—Alpha, Beta, Gamma, and Delta—were provided by NIH RADx^®^ in early December of 2021 (before Omicron samples were available). Specimens were extracted using the Qiagen Viral RNA Mini kit using 140 μL of sample as input and an elution volume of 70 μL. Extracts were tested with all four variant triplexes, as well as with the Biomeme SARS-CoV-2 Dx assay as a control/comparator assay. This testing was performed on a CFX96 instrument using 2 μL of extract per reaction, and testing duplicate PCR reactions of each sample per triplex. Sample identities were blinded to the operators until after data analysis and interpretation was performed.

### Naval Health Research Center (NHRC) clinical sample testing

Clinical samples tested by NHRC consisted of nasal and nasopharyngeal (NP) swabs in either transport media or saline. For sensitivity and specificity testing, we used clinical specimens positive for SARS-CoV-2 of known lineage (determined by the providing lab *via* whole genome sequencing). Ten samples were selected for each variant targeted by the panel except for B.1.351, for which NHRC only had four samples. An additional 20 variants not targeted by the panel were also selected to test for specificity of the variant panels. Viral RNA extraction was performed on 100 μl samples using the Qiagen Viral RNA Mini Kit. Extracts were tested with the CDC 2019-nCoV N1 and N2 assays on an ABI 7500 real-time PCR system (Foster City, CA, USA) to confirm presence of SARS-CoV-2 RNA. Variant panel testing was also conducted on the ABI 7500 using TaqPath 1-Step RT-qPCR Master Mix and the variant panel primers and probes provided by MRIGlobal at a 10X concentration. After initial variant panel testing, high-throughput testing was performed for >1,500 samples using Triplex 4 (the Omicron triplex). During high-throughput testing, all samples were tested with Triplex 4, and in some cases, only samples that were negative for Triplex 4 assays were then tested with the CDC N1 and N2 assays to verify presence/absence of SARS-CoV-2 in the sample.

## Results

### Assay target selection

The Biomeme Franklin^TM^ Real-Time Thermocycler includes three different fluorescence channels, enabling one triplex assay per well. Based on the high prevalence of unique markers among the variant sequences available at the time of assay design, we selected markers to include in each triplex. These markers were either a single nucleotide polymorphism (SNP), multiple SNPs in close proximity, or an insertion or deletion (indel) spanning the probe region. For Triplexes 1 and 2, markers were selected in a way that some are shared among variant lineages and others are unique to a specific lineage to allow for identifying three variants using six markers. For Triplexes 3 and 4, markers were selected for specific identification of a single variant per triplex (Delta and Omicron, respectively). The targeted markers and algorithm for adjudication of results and variant calling are presented in Table 1. Data were obtained from outbreak.info (46).

**Table 1:** SARS-CoV-2 variant genotyping panel.

			**Mutations**																	
			**Triplex 1**			**Triplex 2**			**Triplex 3**			**Triplex 4**								
**WHO designation **	**Number of sublineages **	**Sublineage **	**S: N501Y**	**S: P26S**	**S:Y144-**	**S: H69-/V70-**	**S: E484K**	**S: D215G**	**S:P681R**	**S:L452R**	**S:E156-/F157-**	**S:G142-V143-Y144-**	ins214*	**S:S371L**	**S:S373P**	**S:375F**	S:371F**	**Total lineage/sublineage sequences**	**Total sequences with the set of mutations**	**Percentage of sublineage sequences with the set of mutations**
Alpha	8	B.1.1.7																1,158,196	1,068,792	92
Beta	5	B.1.351																41,849	35,063	81
Gamma	22	P.1																122,431	115,015	93
Delta	250	B.1.617.2																4,312,010	3,869,198	81
Omicron	74	BA.1/BA.1.1																980,269	1,799,390	90/88
		BA.2																341,232	910,646	96

Total sequences as of April 26, 2022: 10,103,411. Source: Outbreak.info Accessed on 04/29/2022 (46). ^*^Outbreak.info can't currently generate statistics for insertions like the S:ins214EPE mutation.

^**^The S:371–375 signature has a different variation in BA.2 and BA.3 (371F) relative to BA.1 (371L), but is still detected by the same assay despite the single-nucleotide difference.

The different colors in the cells represent the corresponding fluorescent channels and probes (Green, FAM; Amber, TEX615; Red, Cy5).

Prevalence and distribution of sequences carrying the markers are also shown in the last three columns of Table 1. The total number of sequences in the database for each variant of concern is displayed, as well as the total number of sequences with a given set of markers. The last column represents the percentage of sequences of the specific sublineage listed in column 2 that carries the set of expected mutations. All triplexes cover 81–96% of the specific sublineages they were designed to detect and distinguish. Since the time of assay design, many new sublineages have emerged, including Omicron sublineages BA.3, BA.4, and BA.5. With the increasing number of sublineages and diversity of markers combined with the convergent evolution of markers, the assignment to sublineages based on just two or three markers has become a challenge. In order to address this issue and to increase the confidence of calls for Omicron sublineages, we expanded the panel to a pentaplex assay described in a later section.

### Assay design

The rationale for assay design was to amplify and detect the target amplicon only if the mutation was present in the template. This was accomplished by designing dual-labeled fluorescent probes that can discriminate between the variant markers and the wild-type sequence, in addition to optimizing the annealing temperature and concentration of the probes. For SNP markers in particular, specificity was accomplished using LNA probe designs. As a first step toward assay design, the primer/probe sequences were examined *in silico* to assess the sensitivity and specificity of the designs against whole genome sequences of SARS-CoV-2 variants obtained from GenBank and GISAID (Table 2).

**Table 2:** *In silico* analyses of the assay signature sequences.

**Lineage:**	**B.1.1.7**	**B.1.351**	**P.1**	**B.1.617.2**	**BA.1**	**BA.1.1**	**BA.2**	**BA.3**	**BA.4**	**BA.5**

**Sequences:**	**95,621**	**738**	**6,443**	**10,507**	**87,849**	**170,416**	**9,040**	**4,370**	**1,174**	**663**
S:N501Y	98%	92%	100%	0.0%	92%	92%	96%	99%	92%	96%
S:P26S	0.1%	0.1%	100%	0.0%	0.0%	0.0%	0.0%	0.0%	0.0%	0.0%
S:Y144del	98%	0.4%	0.2%	0.0%	0.0%	0.0%	0.1%	0.1%	0.0%	0.0%
S:HV69-70del	98%	0.0%	0.0%	0.2%	96%	93%	0.1%	68%	98%	98%
S:E484K	0.2%	93%	97%	0.0%	0.0%	0.0%	0.0%	0.0%	0.0%	0.0%
S:D215G	0.0%	98%	0.0%	0.0%	0.0%	0.0%	0.0%	0.0%	0.0%	0.0%
S:P681R	0.0%	0.0%	0.0%	99%	0.0%	0.0%	0.0%	0.6%	0.0%	0.0%
S:L452R	0.0%	0.3%	0.0%	95%	0.2%	0.0%	0.1%	0.6%	87%	96%
S:EF156–157del	0.0%	0.1%	0.0%	89%	0.1%	0.0%	0.0%	0.3%	0.0%	0.0%
S:S371–375	0.0%	0.0%	0.0%	0.0%	96%	98%	96%	100%	98%	98%
S:GVY142–144del	0.0%	0.0%	0.0%	0.1%	96%	94%	0.0%	100%	0.0%	0.0%
S:ins214EPE	0.0%	0.0%	0.0%	0.1%	79%	86%	0.0%	0.0%	0.0%	0.0%
N:ERS31–33del	0.0%	0.0%	0.0%	0.0%	97%	97%	96%	100%	91%	96%

The *in silico* results indicated very high specificity among the variants of concern, with ≤ 0.6% of sequences for a particular lineage containing any marker not typically associated with that lineage. Predicted sensitivity was >90% for most of the markers, although there were some exceptions for Delta and Omicron lineages. This reduction in sensitivity is generally a reflection of absence of the mutations (as opposed to PCR failure), as there can be differences among sequences of the same lineage and even among sublineages of the same variant. In some instances, there are shared mutations in some lineages or sublineages due to convergent evolution. For example, S:N501Y was initially designed as a shared mutation for the three variants targeted in Triplexes 1 and 2. It is not present in the Delta sequences, but the majority of Omicron sequences carry this mutation. However, this assay failed to produce detection during analytical testing with Omicron (see Table 3) due to mismatches in the forward and reverse primer regions (Supplementary Figure 1). These results were further confirmed by clinical sample testing, during which no positive results were obtained for the S:N501Y assay with Omicron samples (see Supplementary Tables 4, 5). It is noted that when primers were redesigned to incorporate mixed bases at the mismatch sites, the S:N501Y mutation in Omicron could then be reliably detected (unpublished data). Similarly, the S:69/70del mutation is present in both Alpha and Omicron BA.1; however, in the latter, there is an additional SNP in the probe binding region (Supplementary Figures 2, 3). The S:69/70del probe used in Triplex 2 can still detect Omicron albeit at reduced amplitude, while a more Omicron specific probe can be discriminatory between Alpha and Omicron templates (Supplementary Figures 4A–C).

**Table 3:** Testing the specificity of Triplexes 1–4 using SARS-CoV-2 viral gRNA.

**Multiplex**	**Assay**	**A**	**B.1.1.7**	**B.1.351**	**P.1**	**B.617.2**	**B.1.529**
		**wild-type**	**Alpha**	**Beta**	**Gamma**	**Delta**	**Omicron**
Variant Triplex 1	S:N501Y	ND	25.1	28.0	26.2	ND	ND
	S:P26S	ND	ND	ND	24.9	ND	ND
	S:Y144-	ND	22.2	ND	ND	ND	ND
SARS-CoV-2 Dx	Spike	25.3	22.0	24.6	23.0	22.3	24.6
	Orf1ab	25.5	22.1	25.0	23.5	23.6	26.4
Variant Triplex 2	S:H69-/V70-	ND	23.2	ND	ND	ND	27.3
	S:E484K	ND	ND	28.0	26.2	ND	ND
	S:D215G	ND	ND	26.9	ND	ND	ND
SARS-CoV-2 Dx	Spike	24.4	22.5	25.2	23.4	22.4	24.6
	Orf1ab	25.1	23.0	25.7	23.9	22.9	26.4
Variant Triplex 3	S:P681R	ND	ND	ND	ND	24.0	ND
	S:L452R	ND	ND	ND	ND	23.3	ND
	S:E156-/F157-	ND	ND	ND	ND	24.0	ND
SARS-CoV-2 Dx	Spike	26.1	23.9	26.1	24.8	23.8	ND
	Orf1ab	26.2	24.4	26.8	25.2	24.2	ND
Variant Triplex 4	S:371F/373P/375F	ND	ND	ND	ND	ND	24.0
	S:G142-V143-Y144-	ND	ND	ND	ND	ND	23.3
	S:ins214EPE	ND	ND	ND	ND	ND	24.0
SARS-CoV-2 Dx	Spike	25.9	23.8	25.1	24.0	22.4	25.0
	Orf1ab	26.5	24.4	26.0	24.7	23.5	26.3

ND, Not detected.

### Analytical testing of Triplexes 1–4

Initial assay testing and optimization was performed using dsDNA templates and/or *in vitro* transcribed RNA templates harboring the targeted regions (data not shown). Once the assays and reaction conditions were finalized, analytical and clinical testing were performed. Analytical testing of the triplex assays was performed using a serial dilution of live viral stocks after extraction as described in the Materials and Methods section. All assays exhibited the expected results for each lineage. Average Cq values (*n* = 2) are presented for dilutions that produced a Cq value of around 22–25 (Table 3). Comparable values were observed between the diagnostic assays (SARS-CoV-2 Dx: spike and Orf1ab) and the variant specific assays.

**Figure 2:**
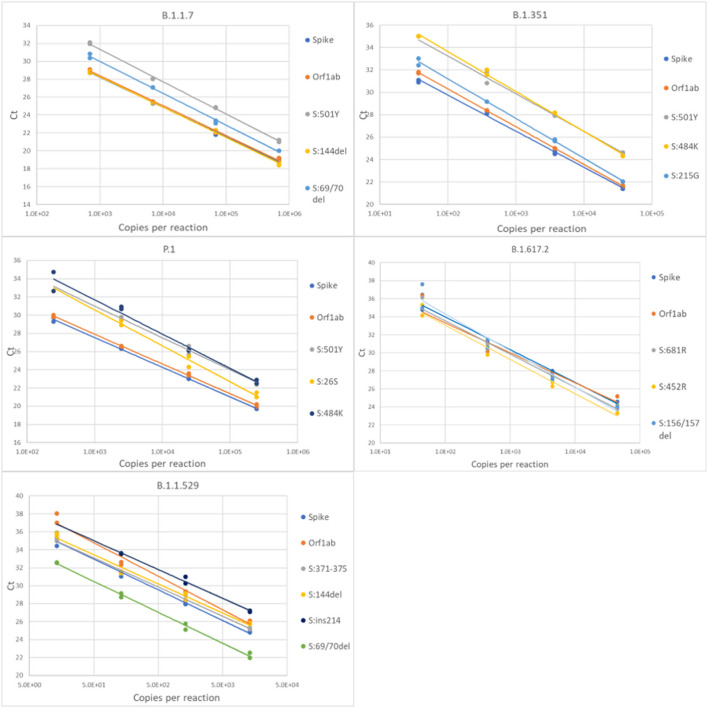
Serial dilution of extracted gRNA of different SARS-CoV-2 variants were used as templates in RT-qPCR reactions. The Cq values are plotted as a function of the concentrations. PCR efficiencies were derived from the slopes of the curves.

For each variant gRNA dilution series and the relevant assays, plots of Cq values vs. viral copies per reaction are presented in Figure 2. The PCR efficiencies are presented in Supplementary Table 6. Amplification efficiencies of all assays with the tested lineages were >80%, with two exceptions (P26S at 79%, and 156/157del at 76%).

### Clinical sample testing

Retrospective testing of the four triplexes using clinical samples was performed as described in the Materials and Methods section. The results are shown in Table 4 and the detailed sample data with Cq values are provided in Supplementary Tables 4, 5. The 64 samples presented in Table 4 are from two different sources: NIH RADx^®^ and NHRC. Overall, the panels have very high sensitivity and specificity (see Supplementary Figures 5A,B). However, in this analysis results of the S:P26S assay in Triplex 1 have been excluded due to indiscriminate signal (and hence false positives) with all SARS-CoV-2 samples analyzed on the ABI 7500 (see Supplementary Figure 5B). Therefore, positive calls for the P.1 lineage are based on only two markers instead of three.

**Table 4:** Positive percent agreement (PPA) and negative percent agreement (NPA) of Triplexes 1–4 for each of the targeted lineages.

**Lineage**	**Samples in the test set**	**True positives**	**False positives**	**Inconclusive (low titer)**	**PPA (%)**	**NPA (%)**
B.1.1.7	15	14	0	1	93.3	100.0
B.1.351	9	9	0	0	100.0	100.0
P.1	15	14	1	1	93.3	98.6
B.1.617.2	15	15	0	0	100.0	100.0
BA.1	10	10	0	0	100.0	100.0

Samples tested with lineages not targeted by the panel: 20.

In addition to the 64 samples from targeted lineages, 20 samples (1 per lineage) from lineages not targeted by the panel were also tested, with only one producing a false positive lineage call; a B.1.621 specimen was falsely identified as P.1 on the basis of having the same two mutations as P.1 when the P26S assay is ignored. This was the only false positive call obtained from the test set of 84 clinical specimens used to assess PPA and NPA of Triplexes 1–4. Difficulty distinguishing amplification from background for the S:P26S assay was seen both in the NHRC clinical sample testing on ABI 7500 systems (Supplementary Figure 5B) and in testing on the Biomeme Franklin^TM^ (**Figure 4**), but was less of an issue during analytical testing and testing of the RADx clinical specimens on the Bio-Rad CFX96.

### Additional clinical sample testing with Triplex 4

Due to the surge in US clinical samples and testing needs for Omicron, beginning in December of 2021, additional testing was performed on the Omicron triplex (Triplex 4). This consisted of dynamic range finding and relative limit of detection (LoD) testing with a clinical specimen, additional sensitivity and specificity testing with an expanded set of samples, and high-throughput testing of >1,500 clinical samples being screened for sequencing.

#### Dynamic range and relative limit of detection (LoD) of Triplex 4

In order to assess the dynamic range and limit of detection of the Triplex 4 panel of assays, a 10-fold serial dilution of a clinical specimen extract was made and PCR was performed as described in the Materials and Methods section. This specimen was known to be SARS-CoV-2 positive and of sublineage BA.1 based on prior PCR and sequencing. Triplicate reactions of Triplex 4 assays were tested for each dilution and the working LoD was determined as the lowest sample level producing 3 of 3 positive results. Working LoD was the same for all three assays in the triplex, with Cq values ranging from 34 to 37 at that level (Table 5). This working LoD was 1:1e5 dilution of the specimen extract, which produced N1 and N2 Cq values of ~18 at the undiluted level.

**Table 5:** Dynamic range testing.

	**S:S371L, S373P, & S375F**			**S:del142–144**			**S:ins214EPE**		
	**Rep 1**	**Rep 2**	**Rep 3**	**Rep 1**	**Rep 2**	**Rep 3**	**Rep 1**	**Rep 2**	**Rep 3**
Undiluted	21.1	21.0	21.3	19.1	19.0	19.1	18.5	18.4	18.6
10^−1^	24.1	24.0	23.9	22.3	22.3	22.2	21.9	21.8	21.6
10^−2^	27.3	27.4	27.1	25.5	25.5	25.4	25.0	25.1	25.0
10^−3^	30.6	30.4	30.6	28.9	28.5	28.7	28.3	28.2	28.3
10^−4^	33.5	33.9	34.1	32.1	32.2	32.3	31.6	31.7	31.8
10^−5^	**36.8**	**37.4**	**37.2**	**34.7**	**35.6**	**34.3**	**34.6**	**34.6**	**34.2**
10^−6^	ND	38.5	ND	37.7	37.4	ND	37.5	37.1	ND
10^−7^	ND	ND	ND	ND	ND	ND	ND	ND	ND

ND, Not detected.

#### Sensitivity and specificity of Triplex 4

**Figure 3:**
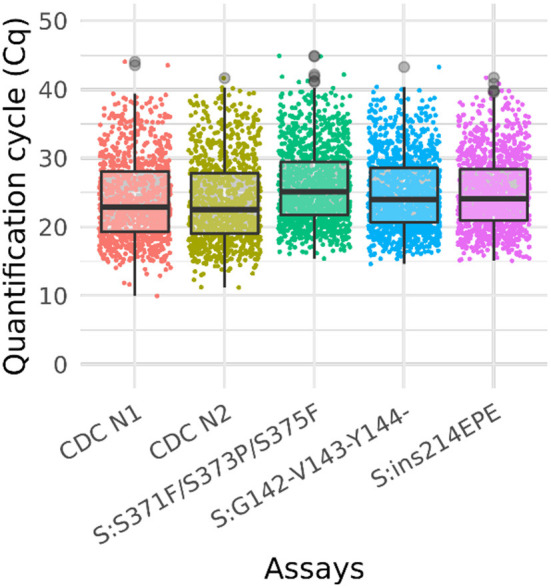
Box plot of Ct values of omicron sample testing results. Cq values for clinical samples tested with Triplex 4 and the CDC N1 and N2 assays. The total number of samples is 1,548. The positive sample counts for each assay are: CDC N1 (1,013), CDC N2 (1,014), S:371–375 (1,255), S:142–144del (1,213), and S:ins214 (1,189). Gray circles represent outliers. Not all samples were tested with the CDC assays.

We further assessed sensitivity and specificity of the Triplex 4 using newly obtained clinical samples. Twenty of 20 Omicron (BA.1) clinical specimens tested positive for all three Omicron assays, including three specimens with “failed” sequencing results due to lower titers. The Cq values of CDC N1 and CDC N2 assays ranged from ~16–30 for these samples. Fourteen of 14 non-Omicron specimens (all of different lineages; see Supplementary Table 5 for lineages) were negative for all three Omicron assays and positive for the CDC N1 and N2 assays, with Cq values of 22–30. Based on these data for sequence-confirmed samples, the sensitivity and specificity were 100% for all three assays in the panel. Ten samples without sequence data were also tested with Triplex 4. Of these ten unknown samples, eight were positive for all three assays, and two were negative. Of the two negative samples, one was positive for the CDC N1 and N2 assays (suggesting it contained a non-Omicron SARS-CoV-2 lineage) and the other one was negative for the CDC assays. Cq data for all samples are summarized in Figure 3 and Supplementary Table 7.

#### High-throughput clinical sample testing with Triplex 4

A total of 1,548 clinical samples were tested with Triplex 4. Of these, 1,255 (81%) were positive for one or more of the Omicron triplex assays. The sample and Cq values of these samples are presented in Supplementary Table 8 and the results are shown in the box plot (Figure 3).

### Performance assessment of all triplexes on the Biomeme Franklin

**Figure 4:**
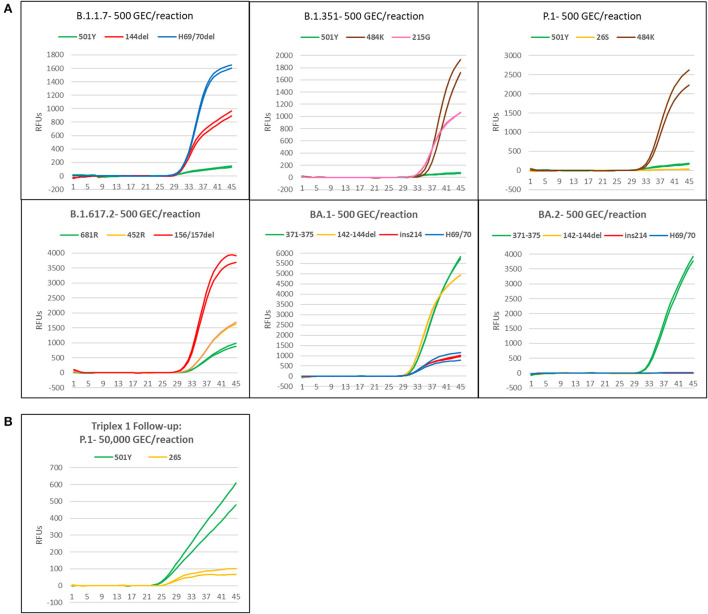
Amplification plots of triplexes 1–4 on the Biomeme Franklin^TM^ device. **(A)** Plots of templates with 500 genomic copy equivalents of different variants. **(B)** Amplification plot of P1 template at 50,000 copies.

Synthetic gRNA obtained from Twist Biosciences for six lineages (B.1.1.7, B.1.351, P.1, B.1.617.2, BA.1, and BA.2) were used to test performance of the variant triplexes on Biomeme Franklin^TM^ devices. The Biomeme Franklin^TM^ is a handheld, battery-powered nucleic acid testing device with 9 wells and 3-color detection that is designed for field portability in low-resource, far-forward environments (39). Negative controls were also included for each assay/run and consisted of water-only NTC reactions, as well as synthetic gRNA from the wild-type (A) lineage. We initially tested each lineage at a concentration of 500 genome equivalent copies (GEC) per reaction. At this level, all assays produced easily discernable amplification for all expected mutations in each lineage, with two exceptions: the S:P26S and S:N501Y assays in which Triplex 1 yielded only dim amplification curves that were not consistently detected (Figure 4A). When Triplex 1 was retested at a 100-fold higher concentration (50,000 GEC per reaction), these assays did produce positive results, but amplification curves remained dim (Figure 4B). These results are consistent with other results obtained on the Bio-Rad CFX96 and ABI 7500, with the S:P26S and S:N501Y assays producing dimmer curves and demonstrating less sensitive detection (higher LoDs) than the other assays in the panel, nonetheless detectable. All NTC reactions and other expected negatives were negative across all runs.

### Omicron pentaplex analytical testing

Omicron pentaplex testing was performed using serial dilutions (50 to 50,000 copies per reaction) of gRNA for BA.1 and BA.2, and of gBlocks for BA.1.1, BA.3, BA.4, and BA.5 due to unavailability of gRNA for those lineages. Wild-type gRNA was used as a negative control. For comparison, these serial dilutions were also tested with Triplexes 1–4. All results were as expected based on *in silico* predictions and known variant mutation profiles according to outbreak.info (40). For positive samples, average Cq values (*n* = 2) are provided for the 50 copies per reaction test level (Table 6). Use of either the Omicron pentaplex or a combination of Triplexes 2–4 allowed for discrimination among BA.1/BA.1.1, BA.2, BA.3, and BA.4/BA.5, whereas use of only the original Omicron triplex (Triplex 4) would not have allowed for discrimination of BA.2 from BA.4 and BA.5. The BA.4 and BA.5 sublineages (as well as some sublineages of BA.2) also have an additional marker of clinical significance, S:L452R, which is part of Triplex 3 and can be used for further discriminatory power (47).

**Table 6:** Inclusivity/exclusivity testing with an expanded omicron panel.

**Multiplex**	**Assay**	**A (wild-type)**	**BA.1 gRNA**	**BA.2 gRNA**	**BA.1.1 gBlock**	**BA.3 gBlock**	**BA.4 gBlock**	**BA.5 gBlock**
Triplex 1	S:N501Y	ND	ND	ND	ND	ND	ND	ND
	S:P26S	ND	ND	ND	ND	ND	ND	ND
	S:Y144-	ND	ND	ND	ND	ND	ND	ND
Triplex 2	S:H69-/V70-	ND	33.0	ND	34.4	33.5	32.5	33.5
	S:E484K	ND	ND	ND	ND	ND	ND	ND
	S:D215G	ND	ND	ND	ND	ND	ND	ND
Triplex 3	S:P681R	ND	ND	ND	ND	ND	ND	ND
	S:L452R	ND	ND	ND	ND	ND	33.2	32.8
	S:E156-/F157-	ND	ND	ND	ND	ND	ND	ND
Triplex 4	S:371F/373P/375F	ND	32.0	33.6	34.0	32.9	32.4	33.3
	S:G142-V143-Y144-	ND	32.3	ND	33.9	33.5	ND	ND
	S:ins214EPE	ND	33.7	ND	34.4	ND	ND	ND
Omicron Pentaplex	S:371F/373P/375F	ND	35.6	35.0	35.8	34.9	34.3	35.0
	S:G142-V143-Y144-	ND	34.5	ND	34.4	34.3	ND	ND
	S:ins214EPE	ND	34.7	ND	33.5	ND	ND	ND
	S:H69-/V70-	ND	34.8	ND	36.0	34.9	36.1	36.3
	N:E31-R32-S33-	ND	35.2	35.9	34.1	34.0	34.4	34.6

ND, Not detected.

## Discussion

The emergence of variants with phenotypic attributes may have impacts on the effectiveness of vaccines, therapeutics, and diagnostics. The resulting mutant proteins can lead to diminished molecular recognition for vaccine and therapeutic interventions (22–25). In regard to diagnostics, signature erosion is a term used for defining the mutational changes in molecular assay signatures such as primer/probe binding sites (48, 49). The term “signature erosion” is used here to signify potential false positive or false negative results in molecular assays due to mutations in the PCR primers/probe/amplicon target sequences (PCR signatures).

In this study, we describe a panel of multiplex RT-qPCR assays targeting a set of mutations across the major SARS-CoV-2 variants of concern; viz., Alpha, Beta, Gamma, Delta, and Omicron for use in a point-of-care PCR device as well as other standard PCR platforms. The Omicron triplex (Triplex 4) can distinguish among Omicron sublineages BA.1, BA.2, and BA.3, and the Omicron pentaplex can further delineate BA.4 and BA.5. We determined a sensitivity and specificity of >90% for these panels with sequence-confirmed SARS-CoV-2 clinical specimens. Based on these data, we posit that these panels could be valuable tools to fill variant testing needs and serve as a screening test to inform selection/prioritization of samples for whole genome sequencing. One of the drawbacks of these panels is that in the current configuration, the panels are used as non-Dx assays because of the cost and time involved in Dx development and are meant to be used as Research Use Only (RUO) assays. The general strategy is to test clinical samples using the Biomeme EUA Dx assay first, followed by screening for variants using the RUO genotyping panel. As of October 2022, there are no EUA SARS-CoV-2 genotyping RT-qPCR assays, although there are a couple with a sequencing component as part of the assay (4).

There are several distinct advantages to using SARS-CoV-2 variant genotyping PCR assays as a screening tool. (1) PCR is extremely less complex, cheaper and faster compared to sequencing, meaning a PCR panel could be a better alternative for rapid identification and tracking of variants (50). (2) A genotyping PCR panel could be very useful in resource-limited settings, since sequencing is not done at the same level in all locations/countries, and many laboratories do not have the infrastructure or expertise to perform sequencing (51). (3) Obtaining whole genome sequence data from clinical samples is very much dependent on viral load; reported PCR Cq cutoffs for obtaining full-length genome sequences range from ~25–33 (52, 53). A PCR panel could fill this gap by providing genotype information for numerous samples that cannot be sequenced due to low viral load as reflected by high Cq values (31). (4) A variant PCR panel can be used as an efficient and cost-effective screening assay to prioritize samples for sequencing, conserve resources, and minimize over/under-sampling of certain lineages for sequencing (54). (5) Mutation-specific PCR amplification can be a more precise option than a proxy based on assay failure, such as the well-known S gene target failure (SGTF). Unlike SGTF, in which negative results are used as a proxy to indicate presence of variants like Alpha and Omicron BA.1, this variant PCR panel comprises specific assays that detect only the mutant template and not the wild type, so variant detection is based on positive results rather than a lack of results. (6) During the course of the pandemic, multiple “discrete” waves of variants emerged. Hence, unique variant panels can be very useful to detect variants with no ambiguity when there are multiple variants in the community; the same is true for mixed infections (55). (7) Wastewater testing is a good predictor of impending community prevalence, with detection of new variants a few days to a week in advance of community prevalence (56), but sequencing wastewater samples to monitor the spread of genotypes is problematic/difficult due to sample quality and the presence of many different strains in the same sample, often at low-titer (57). Thus, a highly sensitive PCR panel that can detect variant markers would be ideal for wastewater monitoring (58). Similarly, the variant panel may also be a useful, cheaper alternative to sequencing and *in vitro* diagnostic (IVD) PCR assays for other types of environmental/non-clinical testing (59) (e.g., contamination/decontamination verification) (60), as RUO assays are typically less expensive than IVD assays. Although not the focus of this study, very limited preliminary studies have also been conducted using our RT-qPCR assays to test for SARS-CoV-2 variants in wastewater samples (unpublished data).

There are of course some limitations of using RT-qPCR panels compared to other approaches such as sequencing. The panels were designed in a way to detect specific variants using a combination of mutations indicative of the variant in the sample. However, these panels may not be useful to detect newly emerging variants with mutations that are not targeted by the panels. Similarly, these panels may not be useful for detecting recombinant variants, since these are potentially chimeras between variants. Also, convergent evolution of the same mutations in multiple lineages may also confound the accurate adjudication of PCR results and assignment of variants present in a sample. In the long run, it would be advantageous to develop assays targeting specific mutations and customize panels depending on the mutations for a given lineage or sublineage in question, and for recombinant lineages (e.g., PCR targeting the junctions or recombination breakpoints). The problem with such a flexible multiplexing approach is that there may not be enough validation data on the performance of a custom triplex with a given set of mutations, unless such data are generated during assay development. In that case, triplex combination testing will become enormous depending on the number of mutations. Hence, the panels described here were designed, tested and validated to detect these specific variants only, and may not work for other variants with a different constellation of mutations, or for recombinant variants. Also, the variants appeared in distinct waves during the course of the pandemic and got replaced by the next wave in a very short time frame. In some cases, the assay development, testing and validation took longer than the time it took for wave replacement, so when the new triplex was ready to be tested in clinical samples, the variant had already been replaced by a newer variant with a new set of mutations. Such an “arms race” between variant emergence and assay development is a challenge that needs to be addressed so that new panels do not become obsolete even before they can be fully developed and verified. Thus, the best approach may be to have assays for different mutations tested and ready to incorporate into customized triplexes, rather than targeting specific lineages that may rise and fall quickly.

Despite these limitations, these variant panels can help fulfill the need for rapid identification of the SARS-CoV-2 variant present in a sample, leading to quick decision making with respect to public health measures as well as treatment options for individuals, especially when sequencing is not an option. In some areas—for instance, many regions in Africa—access to sequencing is extremely limited, while PCR is much more widely available (61, 62). Although progress has been made toward globalizing access to sequencing technology, there are still significant disparities among regions and countries. One reason for this is the significant capability standup costs, including the high cost of next-generation sequencers, accessory lab equipment, data storage and analysis infrastructure, and non-equipment capability standup costs like staff training, protocol development, and method verification studies (63). In addition to the standup costs, another hurdle to broad/universal application of sequencing is the relatively high cost per sample of whole genome sequencing relative to PCR and other nucleic acid amplification tests (NAATs). The RT-qPCR triplexes we developed cost ~$2.83 per reaction in the “wet” (non-lyophilized) format we used for high-throughput testing of clinical specimens, whereas per-sample reagent costs for non-targeted whole genome sequencing are often over $100 depending on instrument and samples-per-run. Although it is possible to achieve per-sample sequencing reagent costs under $10 when using a targeted amplicon sequencing approach and combining a high number of samples (96 or more) on the same sequencing run (64), PCR is still more economical in most cases, especially after accounting for the cost of labor hours, equipment purchases, and other startup costs. Thus, although whole genome sequencing represents a more comprehensive and informative test, multiplexed genotyping RT-qPCR assays like those developed in this study can be invaluable tools when sequencing is not feasible/practical because of access, cost, or time constraints.

## Data availability statement

The original contributions presented in the study are included in the article/Supplementary material, further inquiries can be directed to the corresponding author/s.

## Ethics statement

Ethical review and approval was not required for the study on human participants in accordance with the local legislation and institutional requirements. Written informed consent for participation was not required for this study in accordance with the national legislation and the institutional requirements.

## Author contributions

Conceptualization: Jv, JS, MN, and SS. Experimental design: Jv, MN, EW, JS, and SS. Methodology and experimental validation: BS, MN, BP, BK, PD, RD, RP, and AF. Formal analysis: BS, MN, BP, BK, PD, and JS. Data curation and visualization: JS and BP. Resources: CM, Jv, and JS. Project administration and supervision: Jv, JS, and SS. Funding acquisition: SS and BG. Writing—original draft preparation: SS, JS, and MN. Writing—review and editing: All authors. All authors contributed to the article and approved the submitted version.

## Funding

Funding for this study was provided by the Department of Defense's Joint Program Executive Office for Chemical, Biological, Radiological and Nuclear Defense (JPEO-CBRND), in collaboration with the Defense Health Agency (DHA) COVID funding initiative for this effort: Development of a Biomeme Variant Panel under the Contract (FA807518D0017) with MRIGlobal, and a Subcontract (867-111084-20) between MRIGlobal and Biomeme, Inc. Work performed by Naval Health Research Center (NHRC) was also funded *via* DBPAO contract (N2016).

## Conflict of interest

Authors BS, MN, and Jv were employed by company Biomeme, Inc. Author EW was employed by Integrated DNA Technologies. The remaining authors declare that the research was conducted in the absence of any commercial or financial relationships that could be construed as a potential conflict of interest.

## Publisher's note

All claims expressed in this article are solely those of the authors and do not necessarily represent those of their affiliated organizations, or those of the publisher, the editors and the reviewers. Any product that may be evaluated in this article, or claim that may be made by its manufacturer, is not guaranteed or endorsed by the publisher.

## Author disclaimer

The content of this publication are the authors' opinions and do not necessarily reflect the views or policies of the U.S. Department of Defense, the U.S. Department of the Army, or the institutions and companies affiliated with the authors, nor does the mention of trade names, commercial products, or organizations imply endorsement by the U.S. Government. Some authors of this work are military service members or federal/contracted employees of the United States government. This work was prepared as part of their official duties. Title 17 U.S.C. 105 provides that copyright protection under this title is not available for any work of the United States Government. Title 17 U.S.C. 101 defines a U.S. Government work as work prepared by a military service member or employee of the U.S. Government as part of that person's official duties.
